# Supplementary figures and images for: Optimising primary molecular profiling in non-small cell lung cancer
Source: PLoS One. 2024 Jul 31;19(7):e0290939. doi: 10.1371/journal.pone.0290939 (PMC11290658; doi:10.1371/journal.pone.0290939)

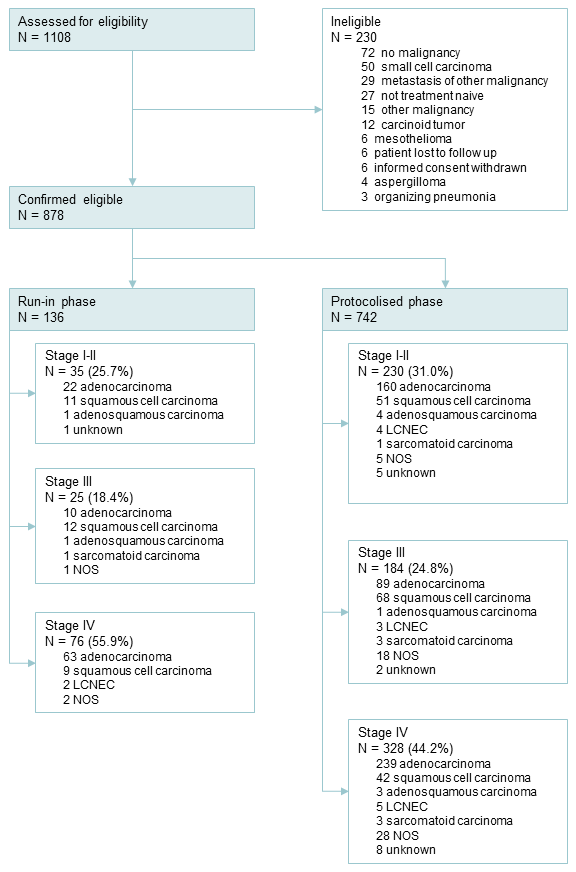

Supplement: S1 Fig — Reasons for ineligibility are shown in the top right panel. This flow chart includes a detailed description of the subtypes of NSCLC that were included in the study. (TIF) [file pone.0290939.s001.tif]
