# Supplementary material for: Optimising primary molecular profiling in non-small cell lung cancer
Source: PLoS One. 2024 Jul 31;19(7):e0290939. doi: 10.1371/journal.pone.0290939 (PMC11290658; doi:10.1371/journal.pone.0290939)
Supplement: S1 Table — The hospitals named in this table have including patients in the Lung cancer Early Molecular Assessment study. All hospitals are located in the Netherlands. (PDF) [file pone.0290939.s002.pdf]

S1 Table. Participating centres. The following hospitals have including patients in the Lung cancer Early Molecular Assessment study. All hospitals are located in the Netherlands.

Netherlands Cancer Institute, Amsterdam
Radboud University Medical Centre, Nijmegen
Noordwest Ziekenhuis Groep, Alkmaar
University Medical Centre Utrecht
Haaglanden Medical Centre, The Hague
Meander Medical Centre, Amersfoort
Onze Lieve Vrouwe Gasthuis, Amsterdam
Tergooi Ziekenhuizen, Hilversum
Flevoziekenhuis, Almere
University Medical Centre Groningen
