# Supplementary material for: Optimising primary molecular profiling in non-small cell lung cancer
Source: PLoS One. 2024 Jul 31;19(7):e0290939. doi: 10.1371/journal.pone.0290939 (PMC11290658; doi:10.1371/journal.pone.0290939)
Supplement: S2 Table — The percentage of patients in whom both EGFR and ALK alteration status was tested is shown. Also, the table shows number and percentages of patients in whom testing was not requested (due to unspecified reasons), and reasons for testing being unfeasible. The numbers correspond to the graphs in Fig 2. In total, 95 out of 136 patients in the run-in phase were tested for both EGFR and ALK alterations (69.9%). This increased to 77.0% in the protocol phase (568/738). Numbers indicate n(%). (PDF) [file pone.0290939.s003.pdf]

S2 Table. Feasibility of *EGFR* and *ALK* testing in tumour tissue. The percentage of patients in whom both *EGFR* and *ALK* alteration status was tested is shown. Also, the table shows number and percentages of patients in whom testing was not requested (due to unspecified reasons), and reasons for testing being unfeasible. The numbers correspond to the graphs in figure 2. In total, 95 out of 136 patients in the run-in phase were tested for both *EGFR* and *ALK* alterations (69.9%). This increased to 77.0% in the protocol phase (568/738). Numbers indicate n(%).

**Any histology**	**Stage I-III**		**Stage IV**	
	**Run-in**	**Protocol**	**Run-in**	**Protocol**
**Total number of patients**	60	414	76	328
EGFR and *ALK* testing status unknown	0	3 (0.7%)	0	1 (0.3%)
Patients tested for *EGFR* and *ALK*	33 (55.0%)	282 (68.6%)	62 (81.6%)	286 (87.5%)
Patients not tested for *EGFR* and *ALK*	27 (45.0%)	129 (31.4%)	14 (18.4%)	41 (12.5%)
EGFR and *ALK* testing not requested	23 (38.3%)	71 (17.3%)	10 (13.2%)	11 (3.4%)
EGFR and *ALK* testing not feasible	4 (6.7%)	58 (14.1%)	4 (5.3%)	30 (9.2%)
Insufficient tumour tissue	3 (5.0%)	36 (8.8%)	4 (5.3%)	20 (6.1%)
No tumour tissue obtained	1 (1.7%)	11 (2.7%)	0	6 (6.1%)
No tumour tissue available at LEMA centre	0	10 (2.4%)	0	1 (0.3%)
Patient died before molecular analysis	0	1 (0.2%)	0	3 (0.9%)

**Non-squamous NSCLC**	**Stage I-III**		**Stage IV**	
	**Run-in**	**Protocol**	**Run-in**	**Protocol**
**Total number of patients**	37	295	67	286
EGFR and *ALK* testing status unknown	0	2 (0.7%)	0	1 (0.3%)
Patients tested for *EGFR* and *ALK*	22 (59.5%)	216 (73.7%)	59 (88.1%)	260 (91.2%)
Patients not tested for *EGFR* and *ALK*	15 (40.5%)	77 (26.3%)	8 (11.9%)	25 (8.8%)
EGFR and *ALK* testing not requested	11 (29.7%)	41 (14.0%)	4 (6.0%)	1 (0.4%)
EGFR and *ALK* testing not feasible	4 (10.8%)	36 (12.3%)	4 (6.0%)	24 (8.4%)
Insufficient tumour tissue	3 (8.1%)	24 (8.2%)	4 (6.0%)	15 (5.3%)
No tumour tissue obtained	1 (2.7%)	8 (2.7%)	0	6 (2.1%)
No tumour tissue available at LEMA centre	0	4 (1.4%)	0	0
Patient died before molecular analysis	0	0	0	3 (1.1%)

**Squamous NSCLC**	**Stage I-III**		**Stage IV**	
	**Run-in**	**Protocol**	**Run-in**	**Protocol**
**Total number of patients**	23	119	9	42
EGFR and *ALK* testing status unknown	0	1 (0.8%)	0	0
Patients tested for *EGFR* and *ALK*	11 (47.8%)	66 (55.9%)	3 (33.3%)	26 (61.9%)
Patients not tested for *EGFR* and *ALK*	12 (52.2%)	52 (44.1%)	6 (66.7%)	16 (38.1%)
EGFR and *ALK* testing not requested	12 (52.2%)	30 (25.4%)	6 (66.7%)	10 (23.8%)
EGFR and *ALK* testing not feasible	0	22 (18.6%)	0	6 (14.3%)
Insufficient tumour tissue	0	12 (10.2%)	0	5 (11.9%)
No tumour tissue obtained	0	3 (2.5%)	0	0
No tumour tissue available at LEMA centre	0	6 (5.1%)	0	1 (2.4%)
Patient died before molecular analysis	0	1 (0.8%)	0	0
