# Supplementary material for: Optimising primary molecular profiling in non-small cell lung cancer
Source: PLoS One. 2024 Jul 31;19(7):e0290939. doi: 10.1371/journal.pone.0290939 (PMC11290658; doi:10.1371/journal.pone.0290939)
Supplement: S3 Table — The table shows the absolute numbers and percentage of successful comprehensive molecular profiling. Also, the frequency of missing requests for profiling (due to unspecified reasons) is shown, and reasons for profiling being unfeasible. The data correspond to the graphs in Fig 3. Numbers indicate n(%). (PDF) [file pone.0290939.s004.pdf]

S3 Table. Feasibility of comprehensive molecular profiling in tumour tissue. The table shows the absolute numbers and percentage of successful comprehensive molecular profiling. Also, the frequency of missing requests for profiling (due to unspecified reasons) is shown, and reasons for profiling being unfeasible. The data correspond to the graphs in figure 3. Numbers indicate n(%).

**Any histology**	**Stage I-III**		**Stage IV**	
	**Run-in**	**Protocol**	**Run-in**	**Protocol**
**Total number of patients**	60	414	76	328
Molecular profiling status unknown	0	2 (0.5%)	0	1 (0.3%)
Completed molecular profiling	31 (51.7%)	249 (60.4%)	52 (68.4%)	259 (79.2%)
Incomplete molecular profiling	29 (48.3%)	163 (39.6%)	24 (31.6%)	68 (20.8%)
NGS and/or ALK test not requested	23 (38.3%)	70 (17.0%)	10 (13.2%)	11 (3.4%)
ROS1 and/or RET test not requested	0	28 (6.8%)	7 (9.2%)	17 (5.2%)
EGFR and *ALK* testing not feasible	6 (10.0%)	62 (15.0%)	5 (6.6%)	35 (10.7%)
Insufficient tumour tissue	5 (8.3%)	40 (9.7%)	5 (6.6%)	25 (7.6%)
No tumour tissue obtained	1 (1.7%)	11 (2.7%)	0	6 (1.8%)
No tumour tissue available at LEMA centre	0	10 (2.4%)	0	1 (0.3%
Patient died before molecular analysis	0	1 (0.2%)	0	3 (0.9%)
Biomarker (ROS1/RET) analysis failed [a]	0	3 (0.7%)	2 (2.6%)	5 (1.5%)

**Non-squamous NSCLC**	**Stage I-III**		**Stage IV**	
	**Run-in**	**Protocol**	**Run-in**	**Protocol**
**Total number of patients**	37	295	67	286
Molecular profiling status unknown	0	1 (0.3%)	0	1 (0.3%)
Completed molecular profiling	20 (54.1%)	202 (68.7%)	51 (76.1%)	241 (84.6%)
Incomplete molecular profiling	17 (45.9%)	92 (31.3%)	16 (23.9%)	44 (15.4%)
NGS and/or ALK test not requested	11 (29.7%)	40 (13.6%)	4 (6.0%)	(0.4%)
ROS1 and/or RET test not requested	0	9 (3.1%)	6 (9.0%)	11 (3.9%)
EGFR and *ALK* testing not feasible	6 (16.2%)	40 (13.6%)	5 (7.5%)	28 (9.8%)
Insufficient tumour tissue	5 (13.5%)	28 (9.5%)	5 (7.5%)	19 (6.7%)
No tumour tissue obtained	1 (2.7%)	8 (2.7%)	0	6 (2.1%)
No tumour tissue available at LEMA centre	0	4 (1.4%)	0	0
Patient died before molecular analysis	0	0	0	3 (1.1%)
Biomarker (ROS1/RET) analysis failed [a]	0	3 (1.0%)	1 (1.5%)	4 (1.4%)

**Squamous NSCLC**	**Stage I-III**		**Stage IV**	
	**Run-in**	**Protocol**	**Run-in**	**Protocol**
**Total number of patients**	23	119	9	42
Molecular profiling status unknown	0	1 (0.8%)	0	0
Completed molecular profiling	11 (47.8%)	47 (39.8%)	1 (11.1%)	18 (42.9%)
Incomplete molecular profiling	12 (52.2%)	71 (60.2%)	8 (88.9%)	24 (57.1%)
NGS and/or ALK test not requested	12 (52.2%)	30 (25.4%)	6 (66.7%)	10 (23.8%)
ROS1 and/or RET test not requested	0	19 (16.1%)	1 (11.1%)	6 (14.3%)
EGFR and *ALK* testing not feasible	0	22 (18.6%)	0	7 (16.7%)
Insufficient tumour tissue	0	12 (10.2%)	0	6 (14.3%)
No tumour tissue obtained	0	3 (2.5%)	0	0
No tumour tissue available at LEMA centre	0	6 (5.1%)	0	1 (2.4%)
Patient died before molecular analysis	0	1 (0.8%)	0	0
Biomarker (ROS1/RET) analysis failed [a]	0	0	1 (11.1%)	1 (2.4%)

[a] in some patients, ROS1 and/or RET tested using FISH failed due to technical reasons
