# Supplementary material for: Optimising primary molecular profiling in non-small cell lung cancer
Source: PLoS One. 2024 Jul 31;19(7):e0290939. doi: 10.1371/journal.pone.0290939 (PMC11290658; doi:10.1371/journal.pone.0290939)
Supplement: S4 Table — Numbers represent n (% of tested in the category), unless stated otherwise. EGFR exon19/21 = currently targetable driver mutations in exon 19 or exon 21. MET ex14del = MET exon 14 skipping mutation. MET amp. = MET amplification. LCNEC–large cell neuroendocrine carcinoma. NOS = not otherwise specified. ca. = carcinoma. TPS = tumour proportion score. ab. = antibody. Percentages in PD-L1 TPS categories represents percentages of patients tested with the indicated antibody (22C3 or any other antibody). (PDF) [file pone.0290939.s005.pdf]

S4 Table. Prevalence of oncogenic driver mutations. Numbers represent n (% of tested in the category), unless stated otherwise. EGFR exon19/21 = currently targetable driver mutations in exon 19 or exon 21. MET ex14del = MET exon 14 skipping mutation. MET amp. = MET amplification. LCNEC – large cell neuroendocrine carcinoma. NOS = not otherwise specified. ca. = carcinoma. TPS = tumour proportion score. ab. = antibody. Percentages in PD-L1 TPS categories represents percentages of patients tested with the indicated antibody (22C3 or any other antibody).

	EGFR (all)	EGFR exon 19/21	EGFR exon 20 ins	KRAS (all)	KRAS G12C	BRAF (all)	BRAF V600E	ERBB2	ALK fusion	ROS1 fusion	RET fusion	MET ex14del	MET amp.
**Patients tested**	691 (78.7%)	691 (78.7%)	691 (78.7%)	690 (78.6%)	690 (78.6%)	689 (78.5%)	689 (78.5%)	687 (78.2%)	555 (63.2%)	462 (52.6%)	259 (29.5%)	713 (81.2%)	690 (78.6%)
**Total prevalence**	78 (11.3%)	66 (9.6%)	5 (0.7%)	238 (34.5%)	104 (15.1%)	24 (3.5%)	12 (1.7%)	13 (1.9%)	14 (2.5%)	8 (1.7%)	7 (2.7%)	15 (2.1%)	9 (1.3%)
**Sex**													
Male	29 (8.1%)	22 (6.1%)	2 (0.6%)	101 (28.2%)	36 (10.1%)	10 (2.8%)	7 (2.0%)	6 (1.7%)	5 (1.6%)	6 (2.3%)	4 (2.5%)	6 (1.6%)	5 (1.4%)
Female	49 (14.8%)	44 (13.3%)	3 (0.9%)	137 (41.3%)	68 (20.5%)	14 (4.2%)	5 (1.5%)	7 (2.1%)	9 (3.6%)	2 (1.0%)	3 (3.1%)	9 (2.6%)	4 (1.2%)
**Age**													
median (years)	66.1	66.1	61.1	66.2	67.7	67.9	69.5	75.4	58.9	68.6	71.7	76.4	66.2
54 or younger	12 (13.0%)	10 (10.9%)	1 (1.1%)	30 (32.6%)	13 (14.1%)	2 (2.2%)	2 (2.2%)	1 (1.1%)	4 (4.8%)	2 (2.8%)	3 (7.5%)	0	0
55 - 64 years	23 (11.5%)	21 (10.5%)	2 (1.0%)	78 (39.0%)	33 (16.5%)	7 (3.5%)	3 (1.5%)	2 (1.0%)	7 (4.3%)	1 (0.8%)	0	1 (0.5%)	4 (2.0%)
65 - 74 years	29 (11.6%)	22 (8.8%)	1 (0.4%)	90 (36.1%)	39 (15.7%)	12 (4.8%)	5 (2.0%)	3 (1.2%)	3 (1.6%)	4 (2.4%)	2 (2.2%)	5 (1.9%)	3 (1.2%)
75 or older	14 (9.4%)	13 (8.7%)	1 (0.7%)	40 (26.8%)	19 (12.8%)	3 (2.0%)	2 (1.3%)	7 (4.7%)	0	1 (1.0%)	2 (3.2%)	9 (5.8%)	2 (1.3%)
**Smoking history**													
current	8 (4.0%)	5 (2.5%)	0	79 (39.9%)	35 (17.7%)	8 (4.0%)	2 (1.0%)	1 (0.5%)	1 (0.6%)	1 (0.7%)	1 (1.3%)	1 (0.5%)	3 (1.5%)
former	24 (10.0%)	17 (7.1%)	5 (2.1%)	80 (33.2%)	36 (14.9%)	9 (3.7%)	7 (2.9%)	5 (2.1%)	5 (2.8%)	3 (1.9%)	0	10 (4.1%)	3 (1.2%)
never	26 (47.3%)	26 (47.3%)	0	5 (9.1%)	2 (3.6%)	2 (3.6%)	2 (3.6%)	1 (1.8%)	6 (14.3%)	3 (9.4%)	2 (12.5%)	4 (7.1%)	1 (1.8%)
unknown	20 (10.2%	18 (9.1%)	0	74 (37.8%)	31 (15.8%)	5 (2.6%)	1 (0.5%)	6 (3.1%)	2 (1.3%)	1 (0.7%)	4 (5.1%)	0	2 (1.0%)

**Stage**													
stage I-II	24 (12.6%)	17 (8.9%)	2 (1.0%)	60 (31.6%)	21 (11.1%)	8 (4.2%)	3 (1.6%)	5 (2.6%)	3 (2.1%)	3 (2.2%)	0	6 (2.9%)	0
stage III	9 (6.4%)	9 (6.4%)	0	34 (24.3%)	18 (12.9%)	2 (1.4%)	0	4 (2.9%)	1 (0.9%)	1 (1.0%)	3 (4.2%)	1 (0.7%)	3 (2.1%)
stage IV	45 (12.5%)	40 (11.1%)	3 (0.8%)	144 (40.0%)	65 (18.1%)	14 (3.9%)	9 (2.5%)	5 (1.1%)	10 (3.4%)	4 (1.8%)	4 (3.5%)	8 (2.2%)	6 (17%)
**Histology**													
adenocarcinoma	75 (14.9%)	63 (12.5%)	5 (1.0%)	211 (42.2%)	92 (18.4%)	21 (4.2%)	11 (2.2%)	13 (2.6%)	13 (3.3%)	8 (2.5%)	4 (2.6%)	13 (2.5%)	8 (1.6%)
squamous cell ca.	3 (2.5%)	3 (2.5%)	0	5 (4.2%)	1 (0.8%)	3 (2.5%)	1 (0.8%)	0	0	0	0	0	0
adenosquamous ca.	0	0	0	3 (42.9%)	2 (28.6%)	0	0	0	1 (14.3%)	0	0	0	0
LCNEC	0	0	0	3 (25.0%)	3 (25.0%)	0	0	0	0	0	1 (12.5%)	0	0
sarcomatoid ca.	0	0	0	3 (42.9%)	2 (28.6%)	0	0	0	0	0	1 (33.3%)	1 (12.5%)	0
NOS or unknown	0	0	0	13 (29.5%)	4 (9.1%)	0	0	0	0	0	1 (4.5%)	1 (2.3%)	1 (2.3%)
PD-L1 TPS (22C3)													
< 1%	23 (52.3%)	19 (50%)	2 (100%)	48 (34.8%)	13 (23.2%)	5 (41.7%)	2 (28.6%)	3 (50.0%)	2 (22.2%)	2 (33.3%)	0	2 (25.0%)	4 (66.7%)
1% - 50%	13 (29.5%)	11 (28.9%)	0	21 (15.2%)	11 (19.6%)	2 (16.7%)	0	1 (16.7%)	3 (33.3%)	1 (16.7%)	3 (60.0%)	2 (25.0%)	2 (33.3%)
> 50%	8 (18.2%)	8 (21.1%)	0	69 (50.0%)	32 (57.1%)	5 (41.7%)	5 (71.4%)	2 (33.3%)	4 (44.4%)	3 (50.0%)	2 (40.0%)	4 (50.0%)	0
PD-L1 TPS (any ab.)													
< 1%	38 (55.9%)	32 (54.2%)	2 (66.7%)	78 (34.8%)	28 (29.8%)	10 (41.7%)	2 (16.7%)	6 (50.0%)	4 (33.3%)	3 (42.9%)	0	4 (33.3%)	5 (71.4%)
1% - 50%	17 (25.0%)	15 (25.4%)	0	44 (19.6%)	18 (19.1%)	2 (8.3%)	0	2 (16.7%)	3 (25.0%)	1 (14.3%)	3 (60.0%)	2 (25.0%)	2 (28.6%)
> 50%	13 (19.1%)	12 (20.3%)	1 (33.3%)	102(45.5%)	48 (51.1%)	12 (50.0%)	10 (83.3%)	4 (33.3%)	5 (41.7%)	3 (42.9%)	2 (40.0%)	5 (41.7%)	0
