# Supplementary material for: Optimising primary molecular profiling in non-small cell lung cancer
Source: PLoS One. 2024 Jul 31;19(7):e0290939. doi: 10.1371/journal.pone.0290939 (PMC11290658; doi:10.1371/journal.pone.0290939)
Supplement: S5 Table — Numbers represent n (% of tested in the category), unless stated otherwise. TPS = tumour proportion score. ca. = carcinoma. LCNEC–large cell neuroendocrine carcinoma. NOS = not otherwise specified. (PDF) [file pone.0290939.s006.pdf]

S5 Table. PD-L1 expression. Numbers represent n (% of tested in the category), unless stated otherwise. TPS = tumour proportion score. ca. = carcinoma. LCNEC – large cell neuroendocrine carcinoma. NOS = not otherwise specified.

	22C3			22C3, SP263, E1L3N, other or unknown antibody		
**PD-L1 TPS**	**<1%**	**1% - 50%**	**> 50%**	**<1%**	**1% - 50%**	**> 50%**
Total prevalence	187 (41.2%)	113 (24.9%)	154 (33.9%)	310 (43.7%)	168 (23.7%)	232 (32.7%)
**Sex**						
Male	103 (43.3%)	61 (25.6%)	74 (31.1%)	174 (45.7%)	94 (24.7%)	113 (29.7%)
Female	84 (38.9%)	52 (24.1%)	80 (37.0%)	136 (41.3%)	74 (22.5%)	119 (36.2%)
**Age**						
median (years)	66.8	64.6	68.5	67.9	65.9	68.4
54 or younger	22 (36.7%)	21 (35.0%)	17 (28.3%)	38 (40.4%)	29 (30.9%)	27 (28.7%)
55 - 64 years	56 (42.1%)	39 (29.3%)	38 (28.6%)	81 (40.7%)	55 (27.6%)	63 (31.7%)
65 - 74 years	69 (39.9%)	35 (20.2%)	69 (39.9%)	117 (44.5%)	54 (20.5%)	32 (35.0%)
75 or older	40 (45.5%)	18 (20.5%)	30 (34.1%)	74 (48.1%)	30 (19.5%)	50 (32.5%)
**Smoking history**						
current	59 (38.8%)	44 (28.9%)	49 (32.2%)	85 (39.9%)	57 (26.8%)	71 (33.3%)
former	73 (44.2%)	37 (22.4%)	55 (33.3%)	108 (44.3%)	55 (22.5%)	81 (33.2%)
never	12 (33.3%)	12 (33.3%)	12 (33.3%)	20 (40.0%)	15 (30.0%)	15 (30.0%)
unknown	43 (42.6%)	20 (19.8%)	38 (37.6%)	97 (47.8%)	41 (20.2%)	65 (32.0%)
**Stage**						
stage I-II	60 (48.8%)	30 (24.4%)	33 (26.8%)	96 (49.7%)	47 (24.4%)	50 (25.9%)
stage III	38 (39.6%)	35 (36.5%)	23 (24.0%)	71 (44.4%)	48 (30.0%)	41 (25.6%)
stage IV	89 (37.9%)	48 (20.4%)	98 (41.7%)	143 (40.1%)	73 (20.4%)	141 (39.5%)
**Histology**						
adenocarcinoma	138 (44.7%)	64 (20.7%)	107 (34.6%)	225 (46.0%)	101 (20.7%)	163 (33.3%)
squamous cell ca.	27 (29.7%)	38 (41.8%)	26 (28.6%)	53 (34.9%)	55 (36.2%)	44 (28.9%)
adenosquamous ca.	0	0	5 (100%)	0	0	5 (100%)
LCNEC	3 (37.5%)	3 (37.5%)	2 (25.0%)	8 (61.5%)	3 (23.1%)	2 (15.4%)
sarcomatoid ca.	2 (33.3%)	0	4 (66.7%)	3 (42.9%)	0	4 (57.1%)
NOS or unknown	17 (48.6%)	8 (22.9%)	10 (28.6%)	21 (47.7%)	9 (20.5%)	14 (31.8%)
