# Supplementary material for: Optimising primary molecular profiling in non-small cell lung cancer
Source: PLoS One. 2024 Jul 31;19(7):e0290939. doi: 10.1371/journal.pone.0290939 (PMC11290658; doi:10.1371/journal.pone.0290939)
Supplement: S6 Table — Results of ddPCR analysis of ctDNA. EGFR exon 19 deletion, EGFR L858R, EGFR T790M and KRAS G12/G13 assessment was performed on a single 4 ml sample of plasma from treatment naïve patients. KRAS mutation analysis included G12A/C/D/R/S/V and G13D mutations. Numbers represent n (% of tested in that category), unless stated otherwise. ҂ two patients had both EGFR exon 19 deletions and T790M in ctDNA. (PDF) [file pone.0290939.s007.pdf]

S6 Table. *EGFR* and *KRAS* mutations in ctDNA. Results of ddPCR analysis of ctDNA. *EGFR* exon 19 deletion, *EGFR* L858R, *EGFR* T790M and *KRAS* G12/G13 assessment was performed on a single 4 ml sample of plasma from treatment naïve patients. *KRAS* mutation analysis included G12A/C/D/R/S/V and G13D mutations. Numbers represent n (% of tested in that category), unless stated otherwise. ‡ two patients had both *EGFR* exon 19 deletions and T790M in ctDNA.

	EGFR ex19del	EGFR L858R	EGFR T790M	KRAS G12/G13
**Feasibility of ddPCR** *(% of samples obtained)*	793 (96.6%)	798 (97.2%)	774 (94.3%)	788 (96.0%)
**Sex**				
Male	3 (0.7%)	6 (1.4%)	1 (0.2%)	47 (11.3%)
Female	13 (3.5%)	10 (2.7%)	3 (0.8%)	53 (14.3%)
**Age**				
54 or younger	6 (6.0%)	1 (1.0%)	2 (2.1%)	14 (14.1%)
55 - 64 years	4 (1.8%)	7 (3.2%)	1 (0.5%)	33 (15.3%)
65 - 74 years	3 (1.0%)	5 (1.7%)	1 (0.3%)	36 (12.1%)
75 or older	3 (1.7%)	3 (1.7%)	0	17 (9.7%)
**Smoking history**				
current	2 (0.9%)	0	1 (0.4%)	38 (16.5%)
former	3 (1.1%)	4 (1.5%)	1 (0.4%)	28 (10.6%)
never	9 (17.0%)	5 (9.4%)	2 (3.8%)	3 (5.8%)
unknown	2 (0.8%)	7 (2.9%)	0	31 (12.9%)
**Stage**				
stage I-II	0	2 (0.8%)	0	5 (2.0%)
stage III	2 (1.1%)	1 (0.5%)	1 (0.5%)	15 (8.1%)
stage IV	14 (3.8%)	13 (3.6%)	3 (0.9%)	80 (22.3%)
**Histology**				
adenocarcinoma	13 (2.5%)	14 (2.6%)	3 (0.6%)	87 (16.5%)
squamous cell carcinoma	1 (0.6%)	2 (1.1%)	1 (0.6%)	2 (1.1%)
adenosquamous carcinoma	0	0	0	0
LCNEC	0	0	0	2 (16.7%)
sarcomatoid carcinoma	0	0	0	1 (12.5%)
NOS or unknown	2 (3.5%)	0	0	8 (14.3%)
