# Supplementary material for: Optimising primary molecular profiling in non-small cell lung cancer
Source: PLoS One. 2024 Jul 31;19(7):e0290939. doi: 10.1371/journal.pone.0290939 (PMC11290658; doi:10.1371/journal.pone.0290939)
Supplement: S7 Table — Analysis of ctDNA for the mutations of interest with ddPCR was performed on a single baseline 4 ml plasma sample. Sensitivity, specificity, positive predictive value (PPV) and negative predictive value (NPV) were calculated using standard contingency tables, including only patients who were tested for EGFR and KRAS in both tissue and blood samples. (PDF) [file pone.0290939.s008.pdf]

S7 Table. Test characteristics of digital droplet PCR. Analysis of ctDNA for the mutations of interest with ddPCR was performed on a single baseline 4 ml plasma sample. Sensitivity, specificity, positive predictive value (PPV) and negative predictive value (NPV) were calculated using standard contingency tables, including only patients who were tested for *EGFR* and *KRAS* in both tissue and blood samples.

Mutation	Stage	n	Sensitivity	Specificity	PPV	NPV
EGFR ex19del	All stages	632	41.4%	99.7%	85.7%	97.2%
	Stage I-II	179	0.0%	100%	n/a	96.6%
	Stage III	127	25.0%	99.2%	50.0%	97.6%
	Stage IV	326	57.9%	99.7%	91.7%	97.5%
EGFR L858R	All stages	637	53.6%	100%	100%	97.9%
	Stage I-II	179	11.1%	100%	100%	95.5%
	Stage III	130	25.0%	100%	100%	97.7%
	Stage IV	328	86.7%	100%	100%	99.4%
KRAS G12A/C/D/R/S/V G13D	All stages	628	46.4%	98.9%	94.4%	81.8%
	Stage I-II	178	8.2%	100%	100%	74.1%
	Stage III	129	35.7%	99.0%	90.9%	84.7%
	Stage IV	321	67.0%	98.1%	94.7%	85.8%
