# Supplementary material for: Optimising primary molecular profiling in non-small cell lung cancer
Source: PLoS One. 2024 Jul 31;19(7):e0290939. doi: 10.1371/journal.pone.0290939 (PMC11290658; doi:10.1371/journal.pone.0290939)
Supplement: S8 Table — The far right column states the reported maximum testing rate of the oncogenes shown under ‘target genes’. Percentage under ‘stage IV’ indicate the percentage of patients with metastatic disease included in the study. The studies were found through a limited PubMed search. This overview is intended to provide reference for the variable testing rates in clinical practice across the world and is not intended as a comprehensive literature review. SCC = squamous cell carcinoma. (PDF) [file pone.0290939.s009.pdf]

**S8 Table. Limited overview of previous studies.** The far right column states the reported maximum testing rate of the oncogenes shown under 'target genes'. Percentage under 'stage IV' indicate the percentage of patients with metastatic disease included in the study. The studies were found through a limited PubMed search. This overview is intended to provide reference for the variable testing rates in clinical practice across the world and is not intended as a comprehensive literature review. SCC = squamous cell carcinoma.

| Study | Type | Period | Region | Patients | Stage IV | Cancer type | Target genes | Test rate |
|---|---|---|---|---|---|---|---|---|
| *Thi, 2020* | Systematic review | 2002-2015 | Global | 52257 | variable | NSCLC | EGFR | 30.9% |
| | Ellis, 2013 | | Canada | 5600 | | | | 31.6% |
| | Pan, 2013 | | USA | 1168 | | | | 11.0% |
| | Sandelin, 2015 | | Sweden | 1646 | | | | 40.0% |
| | Enewold, 2016 | | USA | 1358 | | | | 16.8% |
| | Shen, 2016 | | USA | 5842 | | | | 18.0% |
| | Shinde, 2016 | | USA | 4801 | | | | 13.2% |
| | Gutierrez, 2017 | | USA | 814 | | | | 69.0% |
| | McKeage, 2017 | | New Zealand | 1857 | | | | 27.0% |
| | Xue, 2012 | | China | 606 | | | | 7.8% |
| | Yatabe, 2015 | | Asian Pacific | 22193 | | | | 31.8% |
| | D'Angelo, 2011 | | USA | 1097 | | | | 78.0% |
| | Sequist, 2011 | | USA | 1016 | | | | 53.7% |
| | Boch, 2013 | | Germany | 753 | | | | 73.3% |
| | Choi, 2013 | | Korea | 1503 | | | | 39.0% |
| | Keam, 2013 | | Korea | 1115 | | | | 31.3% |
| | Lim, 2015 | | Canada | 258 | | | | 58.1% |
| | Berg, 2016 | | Norway | 304 | | | | 78.3% |
| | Martorell, 2017 | | Spain | 326 | | | | 73.6% |
| *Lee, 2018* | Retrospective | 2011-2014 | Global | 1440 | 86.3% | any NSCLC | EGFR or KRAS or ALK | 66.0% |
| | | | Italy | 174 | 92.5% | any NSCLC (24.1% SCC) | | 51.1% |
| | | | Spain | 202 | 91.6% | any NSCLC (16.3% SCC) | | 76.2% |
| | | | Germany | 139 | 77.7% | any NSCLC (20.1% SCC) | | 56.1% |
| | | | Australia | 208 | 78.8% | any NSCLC (14.4% SCC) | | 60.6% |
| | | | Japan | 175 | 83.4% | any NSCLC (24.6% SCC) | | 74.3% |
| | | | Korea | 150 | 82.7% | any NSCLC (20.0% SCC) | | 76.0% |
| | | | Taiwan | 217 | 90.8% | any NSCLC (7.4% SCC) | | 85.3% |
| | | | Brazil | 175 | 89.7% | any NSCLC (20.0% SCC) | | 42.9% |

| | | | | | | | | |
|---|---|---|---|---|---|---|---|---|
| *Kuijpers, 2018* | Retrospective | 2013 § | Netherlands | 3195<br>1243 | 100% | non-squamous NSCLC | EGFR, KRAS<br>ALK † | 76,4%<br>56.3% |
| *Kuijpers, 2018* | Retrospective | 2015 § | Netherlands | 3424<br>1325 | 100% | non-squamous NSCLC | EGFR, KRAS<br>ALK † | 81.8%<br>80.9% |
| *Kuijpers, 2017 (poster WCLC)* | Retrospective | 2017 § | Netherlands | 1300 ‡ | 100% | non-squamous NSCLC | EGFR, KRAS<br>ALK † | 81.9%<br>85.6% |
| *Chiang, 2020* | Retrospective | 2011-2017 | USA | 782 | 100% | any NSCLC (12.5% SCC) | EGFR | 71.0% |
| *Colombino, 2019* | Prospective | 2011-2016 | Italy | 1440 | > 83% | lung adenocarcinoma | KRAS, BRAF, MET, ALK | 41.9%† |
| *Sluga, 2018* | Retrospective | 2008-2014 | Netherlands | 2206 | 98.8% | any NSCLC (17.7% SCC) | EGFR, KRAS | 74.4% |
| *Cardarella, 2012* | Prospective | 2009-2010 | USA | 419 | 80% | any NSCLC (2% SCC) | EGFR, KRAS, BRAF, ERBB2, PIK3CA, ALK | 81% |

† percentage of EGFR wildtype patients; ‡ incomplete data; § included only patients with registered tumour tissue in national database.
