# Supplementary material for: Optimising primary molecular profiling in non-small cell lung cancer
Source: PLoS One. 2024 Jul 31;19(7):e0290939. doi: 10.1371/journal.pone.0290939 (PMC11290658; doi:10.1371/journal.pone.0290939)
Supplement: S1 File — This supplement explains the sample- and effect size calculations. (PDF) [file pone.0290939.s010.pdf]

Additional statistical considerations

In the Netherlands 8706 new cases of NSCLC were reported in 2011.(1) The estimated incidence of stage I-II, stage III and stage IV was 25%, 24% and 51%, respectively, these percentages correspond to 2177, 2089 and 4440 patients. Of patients with initial stage I-II disease, 55% were estimated to progress to stage IV disease (n=1197). Of patients with initial stage III disease, 75% were estimated to progress to stage IV disease (n=1567). In total, 7204 out of 8706 new cases would therefore have stage IV NSCLC at some point in the course of the disease and would potentially be eligible for targeted treatment.

Targetable alterations in *EGFR* and *ALK* are present in a fraction of these patients. Based on limited data from other studies, we estimated that the prevalence of *EGFR* and *ALK* alterations was 7.5% and 2.7% in the western European population, respectively.(2,3) Therefore, 736 of 7204 patients were expected to have an *EGFR* or *ALK* alteration. Based on clinical experience we estimated that *EGFR* and *ALK* were tested in 44% of patients, i.e. 321 of 736 expected *EGFR* or *ALK* mutants or 4.5% (321/7204) of all NSCLC patients that would potentially be eligible for targeted treatment.

For the protocolised approach to reach a clinically relevant effect, we postulated that the testing rate of *EGFR* and *ALK* should be higher than 55% (i.e. 403 of 736 patients). Therefore, the detection rate of *EGFR* and *ALK* alterations should be higher than 5.6% (i.e. 403 of 7204 patients).

These parameters were adjusted based on the prevalence of *EGFR* and *ALK* alterations (combined n=92; 10.5%) in the total LEMA cohort. The expected number of patients with an *EGFR* or *ALK* alteration was therefore 755 (10.5% of 7204). With the proposed 55% testing rate 415 of these 755 patients should be identified. This corresponds to a proportion of 5.8% (415 of 7204) of the total number of patient that would potentially be eligible for targeted treatment.

References

1. Integraal Kankercentrum Nederland [Internet]. 2011. Available from: https://iknl.nl/kankersoorten/longkanker/registratie/incidentie

2. Kelly K, Altorki NK, Eberhardt WEE, O’Brien MER, Spigel DR, Crinò L, et al. Adjuvant Erlotinib Versus Placebo in Patients With Stage IB-IIIA Non-Small-Cell Lung Cancer (RADIANT): A Randomized, Double-Blind, Phase III Trial. J Clin Oncol. 2015 Dec;33(34):4007–14.

3. Zhai H, Zhong W, Yang X, Wu Y-L. Neoadjuvant and adjuvant epidermal growth factor receptor tyrosine kinase inhibitor (EGFR-TKI) therapy for lung cancer. Transl lung cancer Res. 2015 Feb;4(1):82–93.
